# Microscopic and mechanical properties of undistributed and remoulded red clay from Guiyang, China

**DOI:** 10.1038/s41598-020-71605-7

**Published:** 2020-10-22

**Authors:** Yanzhao Zhang, Shaoyun Pu, Rita Yi Man Li, Jing Zhang

**Affiliations:** 1 College of Resource and Environmental Engineering, Guizhou University, Huaxi New Campus, Guiyang City, 550025 China; 2 Liyang City Construction Development Group Co., Ltd, Zhuzhou, 213300 China; 3 School of Transportation, Southeast University, Nanjing, 211189 Jiangsu China; 4 Sustainable Real Estate Research Centre, Hong Kong Shue Yan University, Hong Kong, China; 5 College of Geological Engineering and Geomatics, Chang’an University, Xi’an, 710054 Shaanxi China

**Keywords:** Natural hazards, Engineering, Materials science

## Abstract

Unconsolidated-undrained (UU) tests were conducted to investigate the mechanical and morphological properties of undisturbed and remoulded red clay, with the microscopic characteristics determined by scanning electron microscopy (SEM). The microanalysis showed that the red clay aggregate was granular, curved-slice and thin layered and flower-shaped ellipsoid, with X and Y-type cracks and pores in the undisturbed red clay. Moreover, the contact modes of red clay aggregates were point contact, line contact, surface contact and mosaic contact. In addition, the main microstructure red clay was flocculation, honeycomb and pseudosphere structures. The pores in undisturbed soil were arranged in one direction, with no obvious directionality in remoulded red clay. The pore area, perimeter and maximum length of undisturbed red clay were smaller than those of remoulded red clay, with a larger probability entropy, probability distribution index and fractal dimension of pore distribution of undisturbed red clay than remoulded red clay. UU tests showed that the shear strength of undisturbed red clay was higher than that of remoulded red clay.

## Introduction

Red clay refers to the insoluble clay mineral formed by various rocks that make up the earth's crust under the humid and hot climate conditions, its main component is clay mineral^1^. Red clay is brownish yellow–red residual clay formed by carbonate rocks after chemical weathering in moderate temperature and moist climate conditions. Red clay is most widely distributed in Guizhou, Yunnan and Guangxi province as well as in Southern Sichuan, Hunan, Hubei, Guangdong and Jiangxi province in China^2^. The thickness of red clay in China varies greatly and red clay is a special regional soil^2^. Although red clay has a high water content, high void ratio and high plasticity, high bearing capacity, low compressibility and good engineering mechanical properties^3^, so it is used as the filling material for the bearing layer of building foundations. Remoulded red clay and undisturbed red clay are often encountered in engineering. However, the basic mechanical properties of the remoulded red clay and the undisturbed red clay are different due to their different microstructures. Therefore, it is important to analyse the mechanics of remoulded and undisturbed red clay to determine the differences in the microstructure^4^.

As mentioned, the main characteristics of red clay are high natural water content, high liquid limit and large void ratio^5^ but when used as a filling material, the compactability is poor. After compaction, its compressibility is still high^5^. The settlement deformation of the subgrade is large, the deformation stability time is very long, and cracks are produced under drying-wetting cycles^5^. Indeed, the greatest potential danger of subgrade filling with red clay is the generation of deformation and cracks^6^. In the dry season, water evaporation from the soil inside the embankment is rapid, gradually increasing the soil density and producing subsidence and transverse contraction cracks in the embankment^6^. Also, because of its weak resistance to weathering and seepage, the strength of red clay is reduced by the infiltration of rainfall^7,8^. Red clay is composed of kaolinite and illite, with no or a small amount of montmorillonite which determines the swelling shrinkage of red clay^9^. Red clay is a medium expansion potential soil, with a free expansion rate of nearly 80%^3^. The swelling and shrinkage characteristics of expansive soil are the fundamental reasons for engineering damage^10^, and as red clay will expand under drying-wetting cycles, this will affect the safety of the engineering^5^.

Guiyang red clay is a red-brown or yellow–brown regional soil with a high water content, high plasticity, high porosity, high shrinkage and other physical properties^1^, formed as a result of both dissolution-metasomatism and laterization^11^. Dissolution-metasomatism is not only the main factor controlling the properties of red clay but also affects the process of soil evolution in the later stage of soil formation^11^. Due to the rainy climate, Guiyang red clay has a high water content, it is saturated, so the red clay in Guiyang has less expansibility and greater shrinkage^9^.

The microstructure of the soil is related to the sedimentary environment^12^. The microstructure red clay^3^ presents the gelatinous cement and a long strip of flaky particles of clay minerals, larger aggregate in which the cementation strength between aggregates is lower than that between clay particles, and the aggregates with different sizes are cemented to form a larger aggregate by a cementitious substance. The soil is composed of different sized aggregates, with the space between large units filled by small units but there is no clear boundary between units^3^. According to the concept of "aggregate"^13^, aggregate and generalised microstructure model, red clay is a large "aggregate" composed of numerous small "agglomerates" which are gradually stacked and nested, with the formed aggregate mainly exhibiting a granule-flocculent, laminated, skeleton-like and coagulum-like structure^13^ . Zhou et al.^13^ extracted the skeleton form, connecting substance and filling substance of the mentioned-above four structures to form the “grain” structure model of red clay. In this model, the micro-skeleton of red clay comprises several different shaped “grains” with each grain composed of numerous different shaped clay particles cemented together by crystallised strong binding water and numerous fine free iron oxides on the surface of clay particles in the form of particles or films^13^, while the "grain" is connected with other grains by the cohesion of strong and weak bound water, van der Waals forces, mutual electrostatic attraction and colloidal bonding force. Compared with aggregate, the grain is a basic unit formed by mutually cemented clay particles with crystallised strong bound water, free iron oxide and other substances. The shape of grain can be circular, elliptical, layered or flaky, while the aggregate is spherical or ellipsoidal^13^. Clay particle aggregates can be classified as pseudo, water-resistant and true aggregate. The aggregate composed of enriched free iron oxide and free aluminium oxide is water-resistant, i.e. dispersion cannot be achieved by soaking bubbles with water or applying mechanical force^14^. The high strength and low compressibility of red clay are mainly due to the cementation formed by free iron oxide and the special connection between particles^15^.

The microstructure of soil has obvious fractal characteristics^16,17^, with the mechanical properties of soil closely related to its microstructure^18^. The soil microstructure is reflected by some microstructure parameters, including the shape of the aggregate (size, shape and roundness of aggregate), the pattern of the aggregate (direction and arrangement of aggregate), pore characteristics (pore size and distribution), and connectivity (shape and distribution of cementation)^19^. With the development of computer image processing technology, it is possible to study soil microstructure. Recently, microstructure images of soil^20^ have been obtained by SEM^21^ and CT scanning technology^22–25^.

The particle distribution and morphology of red clay exhibit obvious fractal characteristics, with the shear strength increasing with the increased fractal dimension of particle distribution, and there is a good correlation between fractal dimension of particle and shear strength^26^. Regarding Guiyang red clay, most research to date has focused on the macroscopic mechanical properties, more recently, the study of the soil microstructure has also attracted attention. There are few comparative studies on the microstructure of undisturbed and remoulded red clay from Guiyang, therefore, this study aimed to investigate and compare the microstructure of undisturbed and remoulded red clay from Guiyang by triaxial undrained-unconsolidated (UU) and SEM tests.

## Materials and method

### Material

#### Red clay

The soil samples were collected from the engineering geophysical testing site of the School of Resources and Environmental Engineering of Guizhou University in Guizhou Province, China located at 26°26′38″ north and 1,106°39′30″ east in the West Wing of the North–South Guiyang composite anticline and the south of the South–North secondary anticline (Xiaochehe anticline) at an altitude of 1,131.5 m. The geomorphic unit of the study area is corrode-erosion monadnock landforms, with generally flat terrain with little fluctuation. The red clay was formed by the weathering of dolomite from the Triassic Anshun group, is brownish red and brownish-yellow and approximately 8–10 m thick^11^.

#### Basic properties of red clay

**Figure 1:**
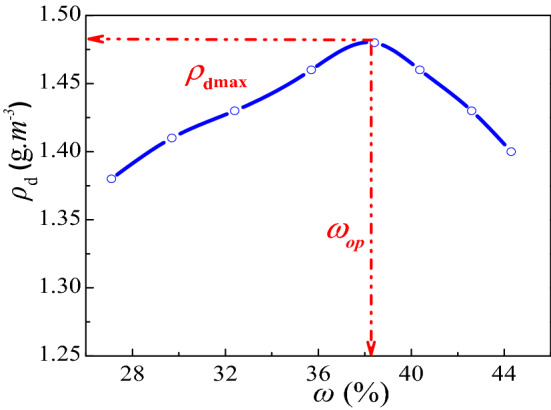
Red clay compaction curve^4^.

**Figure 2:**
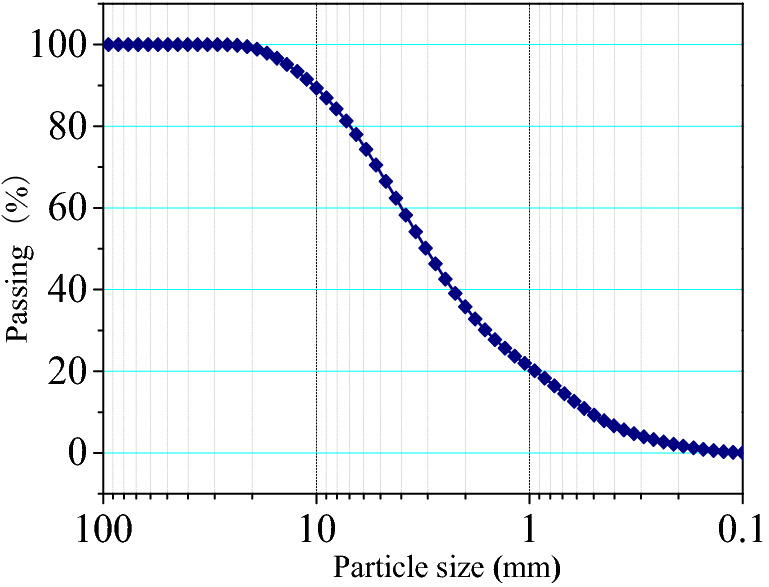
Red clay particle size distribution curve^4^.

The soil was defined as organic clay as per ASTM, D2487^27^ and samples were collected from 4–7 m below ground. The liquid limit was measured as per ASTM D4318-10^28^ and specific gravity was measured as per ASTM D854-10^29^. The natural moisture content and dry density of the red clay was 35.70% and 1.31 g/cm^3^ respectively. The basic physical parameters are shown in Table 1 and the chemical components in Table 2. The red clay from Guiyang was subjected to compaction as per ASTM D698-12^30^ and the obtained compaction curve is shown in Fig. 1, showing that the optimum moisture content of red clay was 38.41%, while the maximum dry density was 1.48 g/cm^3^. In Fig. 1, the dry density $\rho_{{\text{d}}}$ is calculated using the relational expression $\rho_{{\text{d}}} = \frac{\rho }{1 + \omega }$, in which $\rho$ is density of specimens from compation tests, $\omega$ is the water content, which is measured by oven drying method. According to ASTM D422-635^31^, the particle size distribution of red clay was determined by a laser particle size analyser LS 13,320 as shown in Fig. 2, with a void ratio of 1.23 and porosity of 55.16%.

**Table 1:** Physical parameters of red clay.

Dry density (g/m^3^)	Moisture content (%)	Liquid limit^a^ (%)	Plastic limit^a^ (%)	Porosity ratio	Specific gravity^b^
1.46	37.23	76.84	42.16	1.21	2.65

**Table 2:** Chemical components of red clay.

Components	SiO_2_	Al_2_O_3_	Fe_2_O_3_	TiO_2_	K_2_O	SO_3_
Content (%)	58.84	23.96	9.36	2.60	2.00	1.69

### Experimental method

#### Unconsolidated-undrained (UU) test

The unsaturated soil samples were from undisturbed and remoulded red clay, with a diameter of 39.1 mm and 80 mm high. The TSZ series automatic triaxial apparatus (Nanjing soil instrument factory) was used according to the standard geotechnical test method (GB/T 50123-1999). Before the experiment, repeated water injection and water drainage were performed to remove any bubbles in the water inside the pipe, then the UU tests were performed as per ASTM D2850^32^. Throughout the test, the shear rate was controlled at 0.08 mm/min and the test was terminated when the strain reached 20%. The applied confining pressures were 50, 100, 200, 300, and 400 kPa. It should be noted that for specimens with strain hardening, the failure strain $\varepsilon_{1f}$ is defined as 20%, and the deviatoric stress strength is defined as $\sigma_{1} - \sigma_{3}$ value when strain reaches 20%.

#### SEM tests

SEM analysis was performed to investigate the morphology of undisturbed and remoulded red clay using an electron microscope scanner EVO MA 15/LS 15. The test soil sample was cut into small pieces (1 cm^3^) with a thin steel wire with a 0.5 mm deep groove cut in the middle of the sample, then freeze-dried for 24 h. The observed surface was a horizontal profile of soil samples, with samples cut along the direction of soil deposition. Before scanning, the soil sample was gently forced apart from the groove position, and a relatively flat and fresh section selected for testing. The broken soil sample was sprayed with gold to improve conductivity, then placed in the specimen holder and SEM images were collected at different magnifications using a secondary electronic SE probe and backscatter BSE detector.

#### X-ray diffraction (XRD) tests

The XRD test was performed using an X-ray diffractometer X'Pert Powder (PAN Alytical B.V.). The sample was oven dried for 24 h, then ground into fine powder for testing. The scanning speed used was set at ${2}^\circ {(2}\theta {\text{)/min}}$, with a scan range of $2\theta$ (5–90°). The working voltage and current of the diffractometer optical tube were 40 kV and 40 mA, respectively.

## Results and discussion

### Mechanical properties of undisturbed red clay

**Figure 3:**
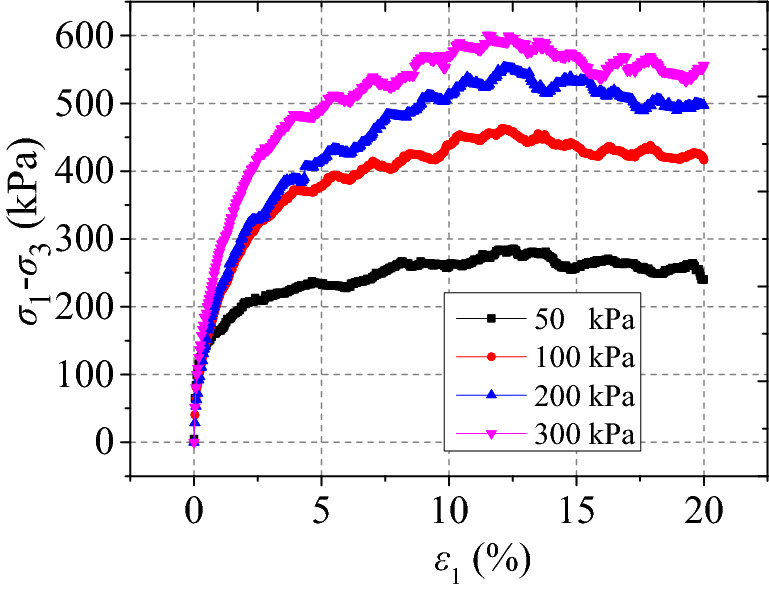
Undisturbed red clay stress–strain curve^34^.

**Figure 4:**
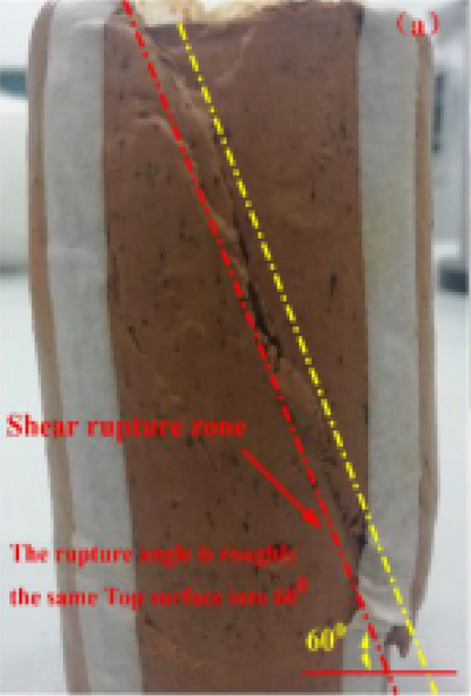
Undisturbed red clay failure modes^34^.

The curves $\left( {\sigma_{1} - \sigma_{3} } \right) - \varepsilon_{1}$ of undisturbed red clay from UU tests are shown in Fig. 3, demonstrating camel humps. The undisturbed red clay exhibited strain-softening behaviour and as the axial strain $\varepsilon_{1}$ increased, the deviatoric stress $\left( {\sigma_{1} - \sigma_{3} } \right)$ initially increased, demonstrating a slow downward trend after the stress reached peak stress, indicating that the clay presented plastic failure. This may be due to the consolidation state and the microstructure of red clay. When the specimen was loaded, the specimen was compacted, thus the pores were small and soil particles were rearranged, hence, the resistance of the soil to external load increased. However, when the external load exceeded the structural yield stress of the red clay, the dilatancy failure of clay occurred (see Fig. 4), with the soil gradually losing its bearing capacity due to the dilatancy.

### Mechanical properties of remoulded red clay

**Figure 5:**
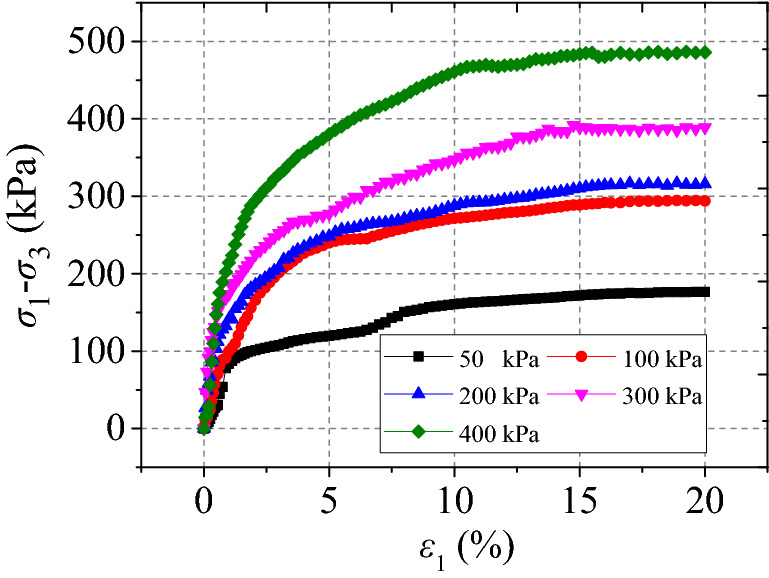
Remoulded clay stress–strain curves^34^.

**Figure 6:**
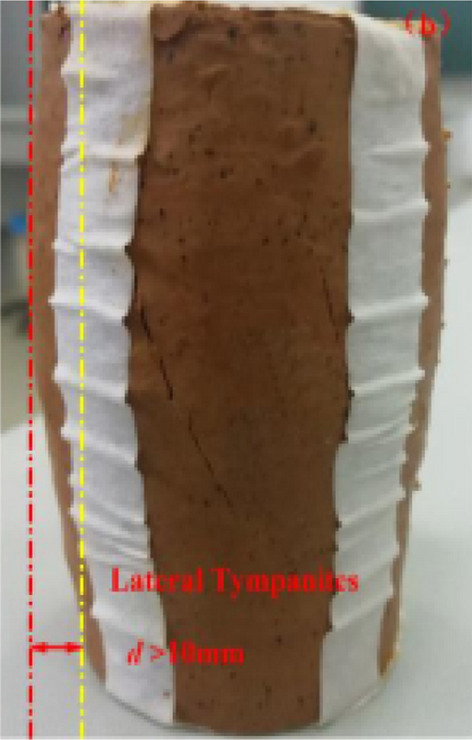
Remoulded clay failure modes^34^.

The relationship $\left( {\sigma_{1} - \sigma_{3} } \right) - \varepsilon_{1}$ curves of remoulded red clay under different confining pressures are presented in Fig. 5, showing strain hardening behaviour, while the trends of curve change under different confining pressures were identical. Moreover, the deviatoric stress $\left( {\sigma_{1} - \sigma_{3} } \right)$ strength increased with the increase in confining pressure, as there was an increase in the initial tangent modulus, possibly due to compression of the large pore size and increasing bonding force between particles with the increased confining pressure. During the initial stage of loading, the deviatoric stress $\left( {\sigma_{1} - \sigma_{3} } \right)$ increased linearly with the increase of axial strain $\upvarepsilon$_1_ with the $\left( {\sigma_{1} - \sigma_{3} } \right) - \varepsilon_{1}$ becoming nonlinear and the soil showed strain hardening and bulging (see Fig. 6). When the confining pressure increased, the strain hardening effect of the soil became more obvious.

**Figure 7:**
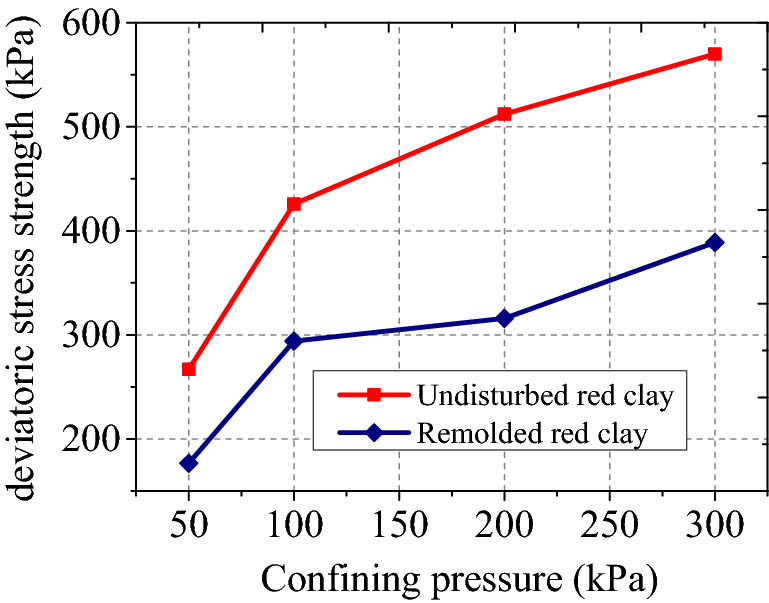
Deviatoric stress strength of remoulded and undisturbed red clay.

**Figure 8:**
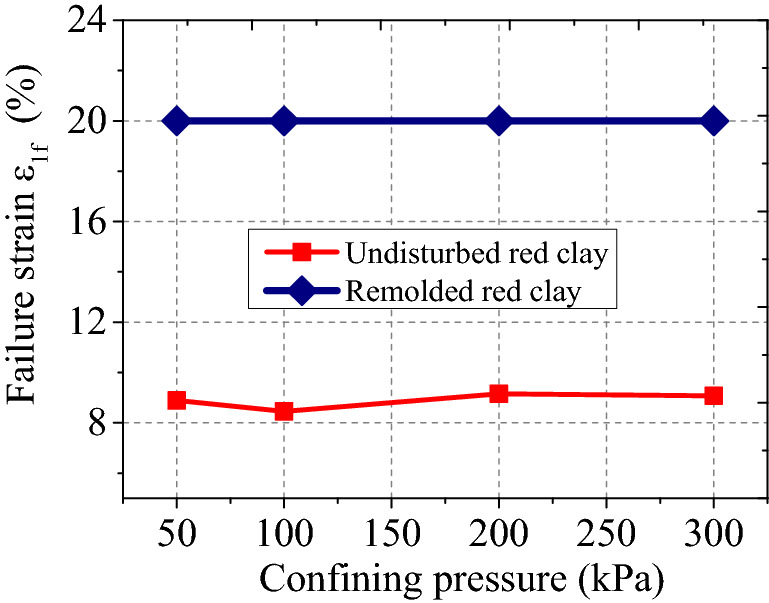
Failure strain of remoulded and undisturbed red clay.

Figures 7 and 8 show the deviatoric stress strength and failure strain of undisturbed and remoulded red clay under different confining pressures. It can be seen from Fig. 7 and 8 that the deviatoric stress strength of undisturbed and remoulded red clay increased with increasing confining pressure, with a lower deviatoric stress strength of remoulded red clay lower than undisturbed red clay. Undisturbed red clay has a primary structure so when the load exceeds the structural yield stress, the original structure is destroyed, resulting in the sudden loss of the bearing capacity of the soil, thereby exhibiting strain-softening behaviour. However, for remoulded red clay, the original structure of the soil has been destroyed before the test, so the particles will rearrange under load, hence the external load exhibits strain hardening. From Fig. 8, the failure strain $\varepsilon_{1f}$ of remoulded clay was larger than that of undisturbed red clay.

### Microstructure analysis

#### XRD analysis

**Figure 9:**
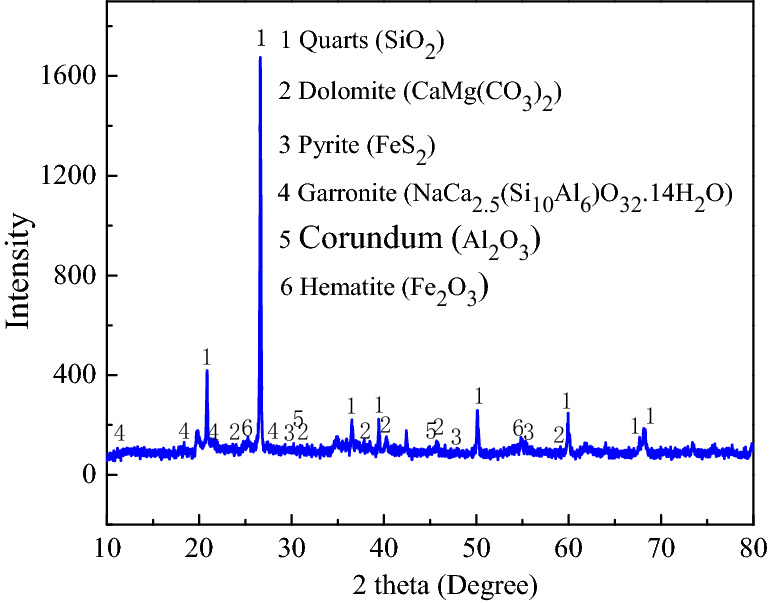
Guiyang red clay XRD diagram^4^.

As shown in Fig. 9, Guiyang red clay mainly contains quartz, pyrite, hematite, Corundum (Table 3), clay minerals consisting of Garronite and dolomite in clay minerals. The existence of dolomite is related to its genesis. The red clay in the study area is formed by the weathering products of dolomite after dissolution-replacement and laterization, so there are a lot of dolomite in the red clay. Pyrite plays a role in cementing the soil particles, whereas hematite gives the soil its characteristic red colour.

**Table 3:** Mineral components of red clay.

Components	Quartz (SiO_2_)	Corundum(Al_2_O_3_)	Hematite (Fe_2_O_3_)	Garronite (NaCa2.5(Si_10_Al_6_)O32·14H_2_O )	Dolomite (CaMg(CO_3_)_2_)
Content (%)	41.7	1.03	2.93	39.5	2.19

#### Micromorphology of undisturbed red clay

##### Aggregate morphology of undisturbed red clay

**Figure 10:**
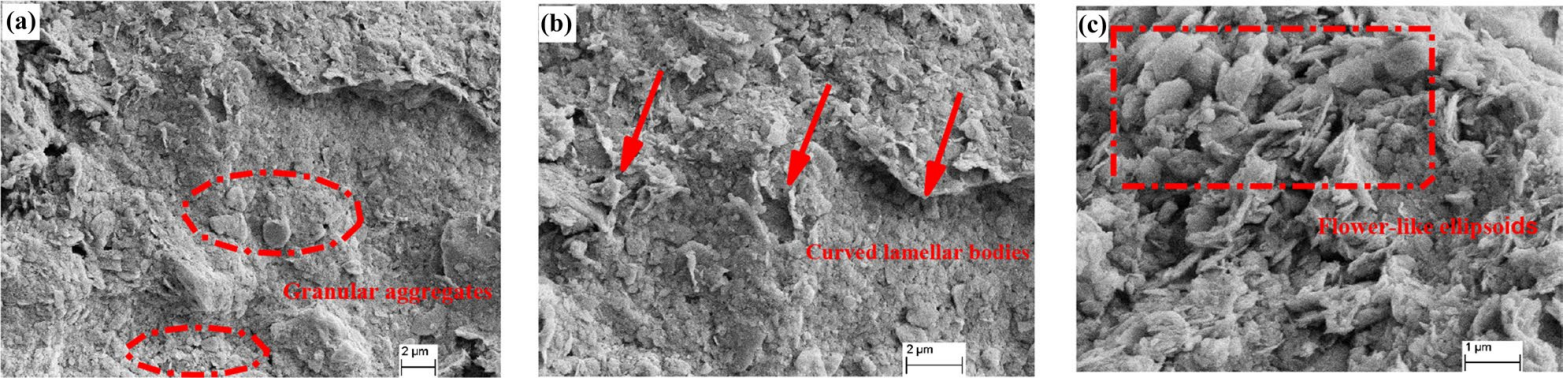
SEM images of undisturbed red clay: (**a**) granular particles, (**b**) curved-slice and thin layer-shape, and (**c**) flower-shaped ellipsoid aggregate.

Figure 10 presents the SEM images of undisturbed red clay, showing granular-shape (Fig. 10a), curved-slice and a thin layer (Fig. 10b), as well as flower-shaped ellipsoid particles (Fig. 13c). The granular aggregate is mainly formed by the aggregation and cementation of crystalline garronite or dolomite, with clear boundaries between particles, a long axis of 6.6 μm, a short axis of 4.8 μm and an area of 30.57 μm^2^. The curved-slice and thin layer aggregates were slightly curled and not very thick, with no obvious boundary between the aggregates due to the parallel cementation of garronite or dolomite crystals with a certain degree of crystallisation. The long axis and short axis of the curved-slice and thin layer particle aggregates with an area of 59.37 μm^2^ were 8.9 and 6.8 μm, respectively. The flower-shaped particles were formed by the crystallised illite radiating outward from a crystallisation centre, with clear boundaries, a long axis of 7.9 μm, the short axis of 6.3 μm and an area of 48.25 μm^2^.

##### Cracks and pores

**Figure 11:**
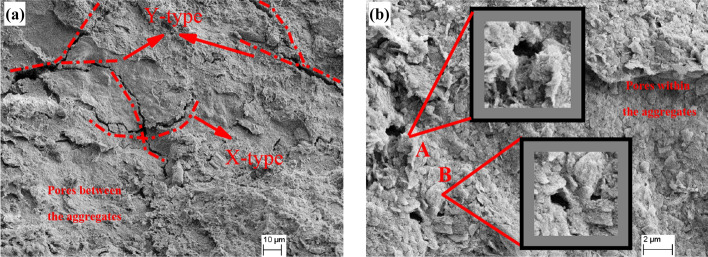
Crack and pore types of undisturbed red clay: (**a**) crack type of undisturbed red clay, and (**b**) internal pores of undisturbed red clay.

Figure 11 shows cracks and pores between or within aggregates of undisturbed red clay. The cracks are classified as X and Y-type (Fig. 11a) and are formed by the dry shrinkage of soil in the natural environment. There were some internal pores (diameter of 3.4 μm and an area of 4.8 μm^2^) between aggregates which are formed when clay particles are cemented to form a soil skeleton in space or contacted mutually in a plane (Fig. 11b). The internal pores (diameter of 2.2 μm and area of 3.6 μm^2^) were composed of pores between the three kinds of aggregates described above.

**Figure 12:**
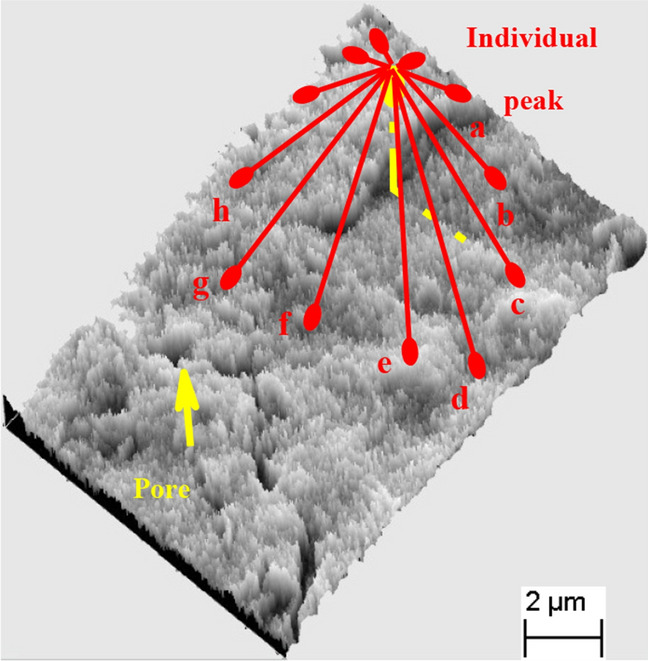
Three-dimensional visualisation of the microstructure.

Figure 12 shows the 3D visualisation of the complex microstructure of undisturbed red clay with an irregular surface profile as well as some cracks and pores. The contact modes between particles were diversified. The structure plane of red clay was almost-flat and randomly rough because the aggregates are pseudospherical, flower-like or irregular.

##### Contact-bonding modes of red clay aggregates

**Figure 13:**
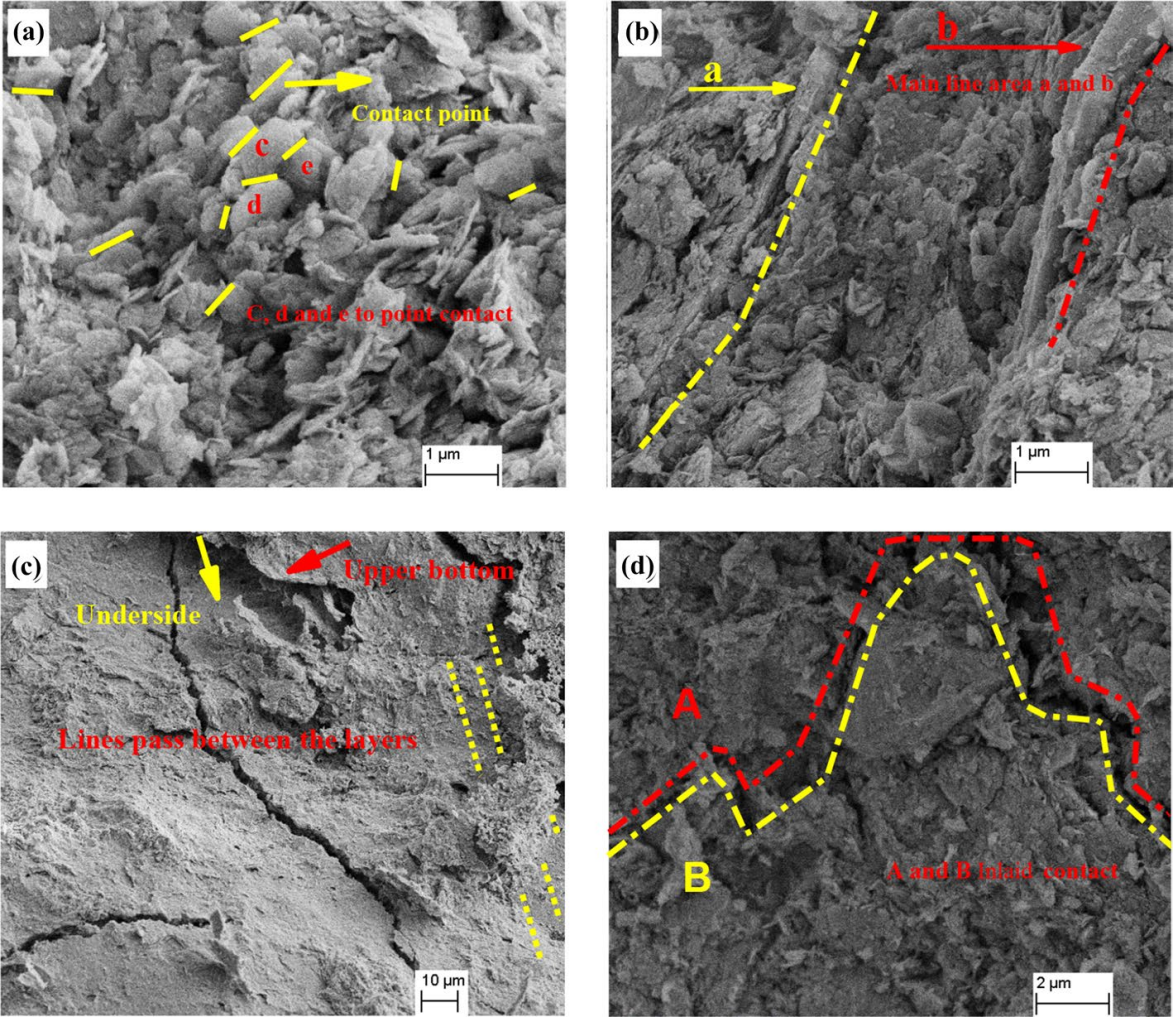
Contact-bonding modes of red clay aggregates: (**a**) point contact, (**b**) line contact, (**c**) surface contact, and (**d**) mosaic contact.

The contact-bonding modes between aggregates are closely related to the mechanical properties of soils and classified as edge-edge, edge-surface, point-surface, and surface-surface contact modes^34,35^. Point-surface contact is not common in nature, while face-surface and edge-edge contact are very common in soils containing garronite or dolomite The edge-edge contact appears in the process of flocculation structure formation. Figure 13 shows the contact-bonding forms of red clay aggregates are point, line, surface and mosaic contact. The mosaic contact was formed because the flat-flocculated soil particles were staggered mutually under the cementation of free iron oxide, and the whole mosaic contact of particles was formed after chemical and mechanical occlusion.

##### Microstructure forms

**Figure 14:**
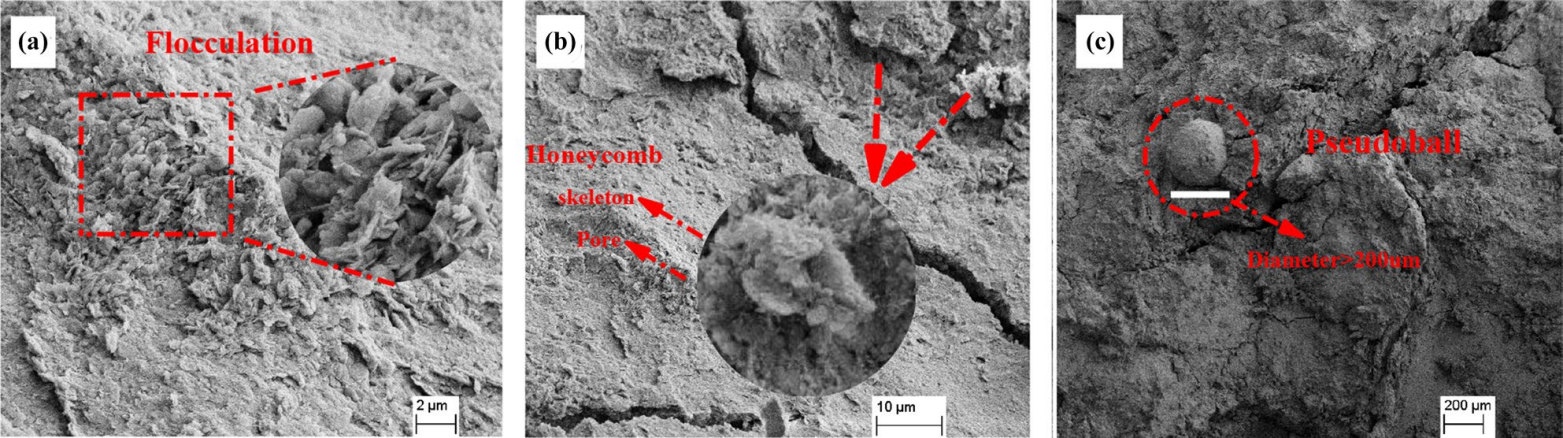
Microstructure forms of undisturbed red clay: (**a**) flocculated, (**b**) honeycomb and (**c**) pseudosphere.

There are nine types of clay microstructures existing in nature: skeleton structure, honeycomb structure, matrix structure, laminar structure, turbulent structure, magnetic domain structure, sponge structure, pseudosphere structure^36^ and flocculated structure^37^. As shown in Fig. 14, undisturbed red clay from Guiyang possessed flocculation, honeycomb and pseudosphere structures, with the main structure being flocculated and honeycomb-like.

#### Comparison of the micromorphology of undisturbed and remoulded red clay

**Figure 15:**
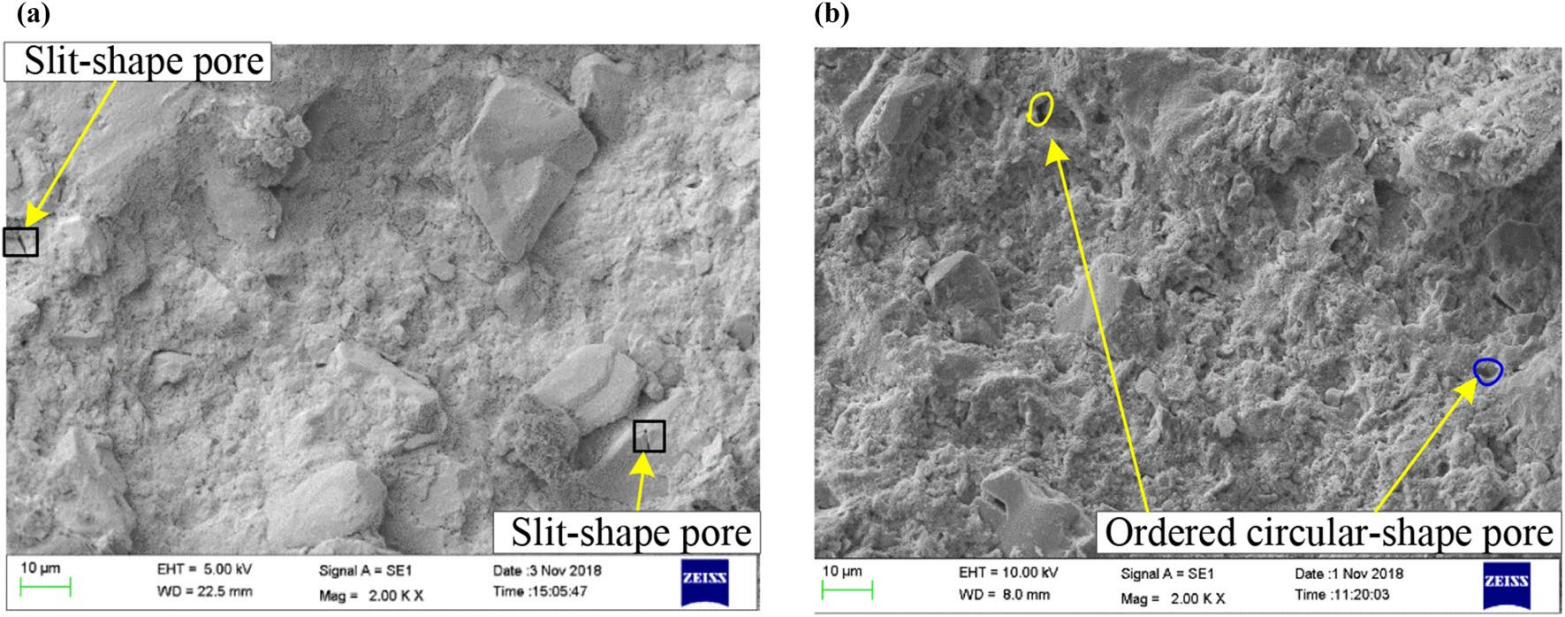
SEM images of Guiyang undisturbed (**a**) and remoulded (**b**) red clay.

The SEM images (2000 × magnification) of Guiyang undisturbed and remoulded red clay are presented in Fig. 15, showing that there are fewer pores in the more compact undisturbed red clay compared to the remoulded clay. Also, the pores in the undisturbed red clay were “slit-shape”, whereas the remoulded red clay pores were “ordered circular-shape”.

**Figure 16:**
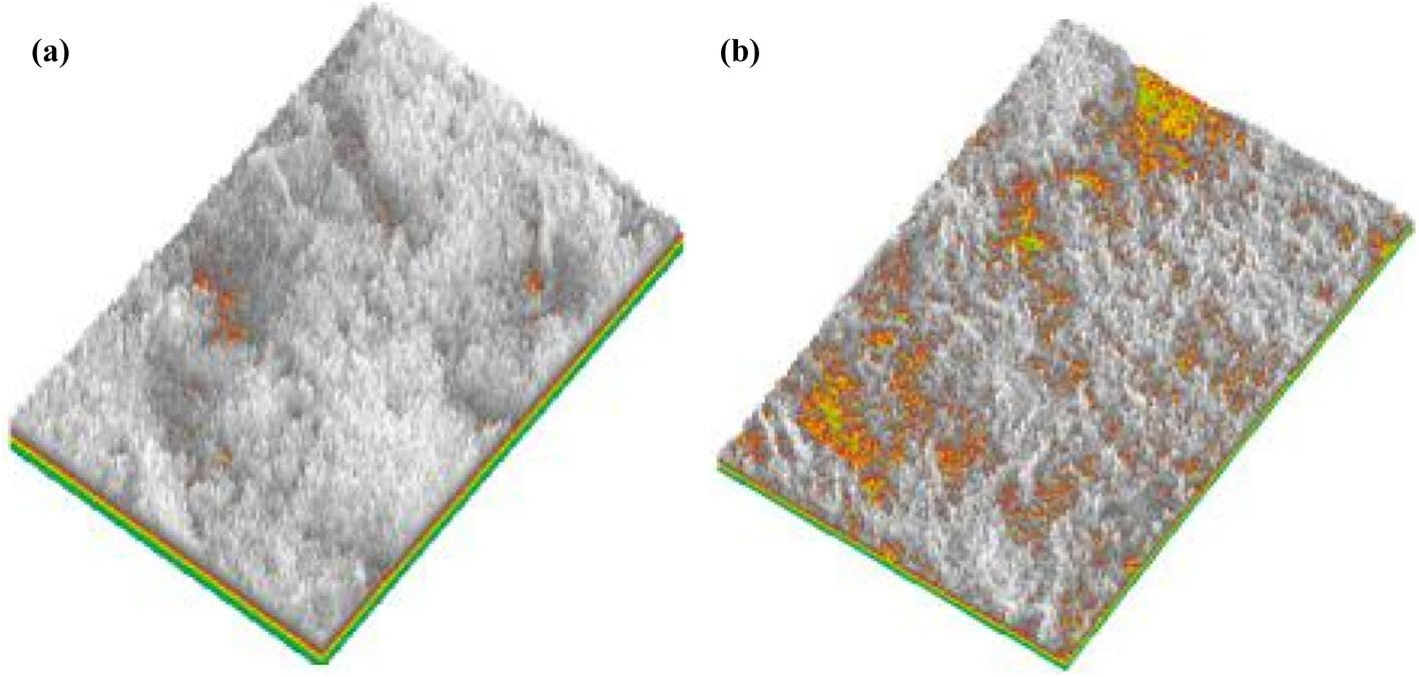
3D visualisation of the microstructure of undisturbed (**a**) and remoulded (**b**) red clay.

The 3D visualisation images in Fig. 16 show that the undisturbed red clay microstructure was more complex with a more irregular surface profile and fewer pores compared to remoulded red clay. Also, the natural honeycomb and flocculation structures of the undisturbed soil were partially destroyed in remoulded red clay. Consequently, some clastic flat clay minerals appeared to overlie the agglomerated matrix surface in direct contact, which decreases the degree of coalescence and aggregation between particles, so remoulded red clay has greater porosity. Hence, undisturbed soil has better mechanical properties in comparison with remoulded red clay under confining pressure, as well as exhibiting strain-softening behaviour in contrast to the strain hardening behaviour of remoulded soil.

**Figure 17:**
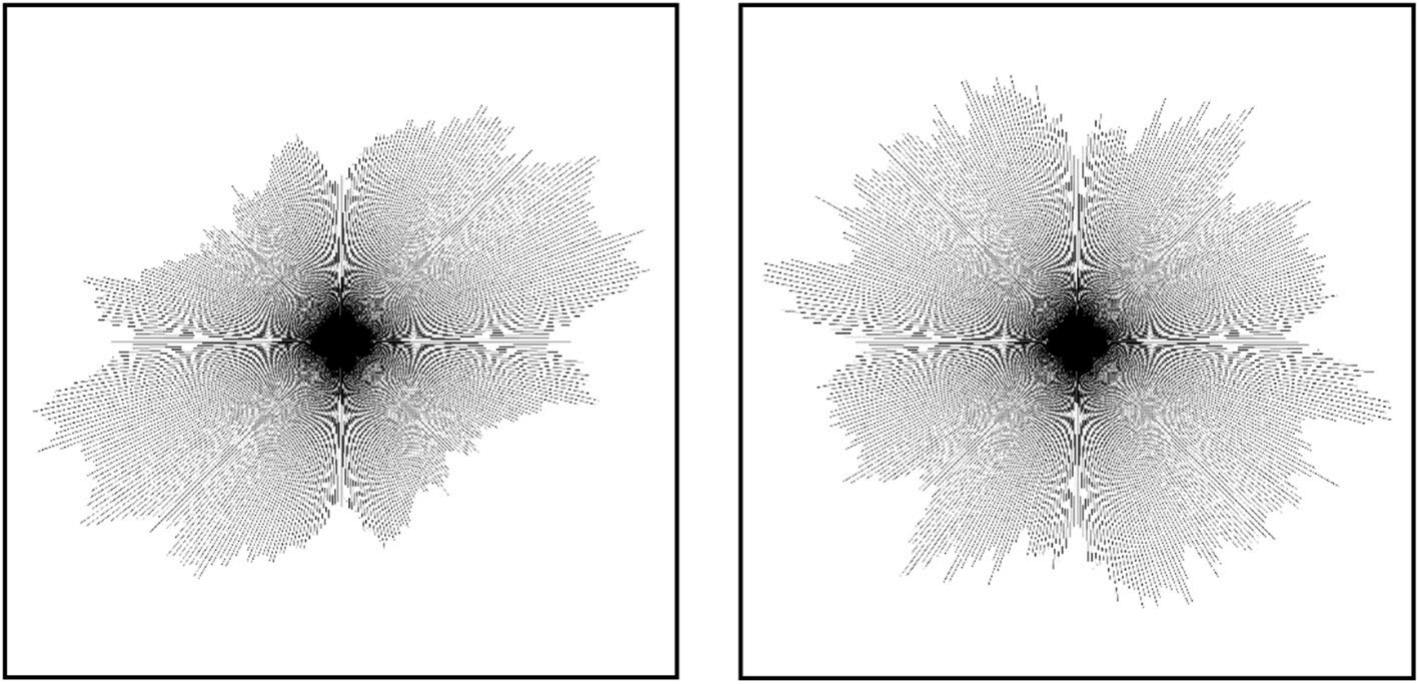
Rose map of the direction of the pore distribution in undisturbed (**a**) and remoulded red clay (**b**).

Figure 17 is the rose map of pore distribution of undisturbed and remoulded red clay, showing that the undisturbed soil presented clear directionality in pore distribution, with all pores distributed in one direction, whereas the pores in remoulded red clay had no obvious directionality. The directionality of pore distribution affects the mechanical properties of soil, thus, pores with no directionality could easily merge to form a larger pore under external load, which would increase the distance between aggregates. If all pores are distributed in one direction, the soil would exhibit good mechanical properties. Consequently, under same confining pressure, the mechanical properties of undisturbed red clay were better than those of remoulded red clay (see Figs. 3 and 4) due to the pore arrangement, as illustrated by the pore microstructure parameters of remoulded and undisturbed red clay discussed later in this article.

### Quantitative analysis of microstructure

The microscopic images of red clay were obtained by SEM using a secondary electron SE probe. For quantitative analysis, the microscopic images were captured using a backscatter BSE detector to investigate the pore microstructure parameters of the undisturbed and remoulded red clay.

#### Optimisation of influencing factors

When SEM images were used to quantitatively analyse the microstructure parameters of soil, some factors such as magnification, threshold value and analysis areas were found to be important, among them, magnification and threshold were the most important. For soft clay, the optimum magnification range for SEM is 600–2000×^38^, however, the optimum magnification range and grey threshold may be different for different soils. Consequently, the influence of magnification and grey threshold on the microstructure analysis were studied in the following sections.

#### Optimisation of magnification

**Figure 18:**
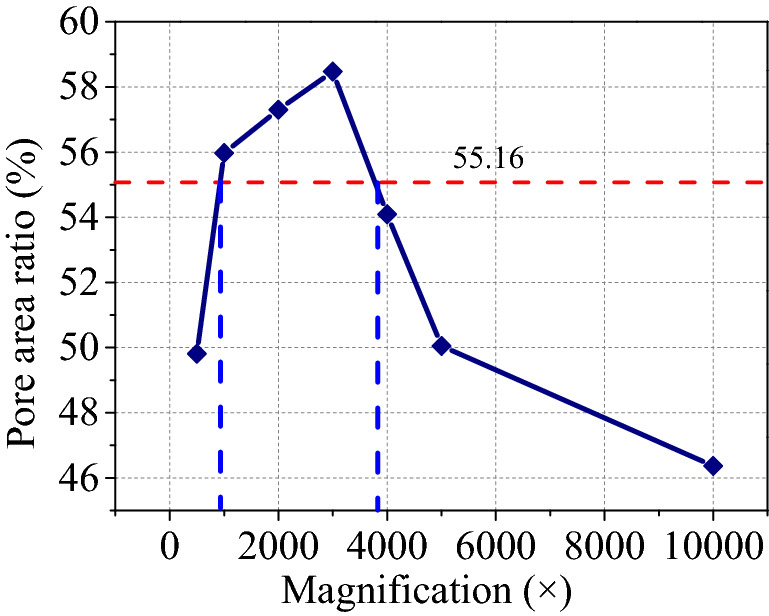
Influence of magnification on the measure of porosity.

To obtain the appropriate magnification of the red clay, it was first scanned under a magnification of 500×, then photographed continuously under magnification from 1,000× to 5,000× with an increment of 1,000×. Besides, the samples were also observed at a magnification of 10,000×. The porosity of all collected SEM images with different magnifications was calculated using image Pro-Plus 6.0 software as shown in Fig. 18. The obtained porosity from SEM images was affected by magnification, when the magnification was less than 2000× or greater than 3,000×, the obtained porosity deviated from the test porosity. The porosity of the soil in nature is not affected by magnification, rather magnification will affect the calculations from SEM images. Therefore, in the micro quantitative analysis, it is very important to choose the appropriate image magnification, for example, the maximum porosity was found to be 58.47% at a magnification of 3,000×, with the obtained porosity at magnifications of 500× and 1,000× of 49.81% and 46.37%, respectively. The obtained porosity at the magnification of 2000× and 1,000× were 17.39% and 26.10%, larger than those at the magnification of 500× and 1,000×, respectively. Consequently, for red clay, the magnification also has a significant effect on the obtained porosity from SEM images.

From Fig. 18, when the magnification of image was between 1,500× and 4,000×, the obtained porosity was close to the test porosity, however, when the magnification was less than 1,500× or lager than 4,000×, the SEM images could not be used to microstructure parameters of red clay. Consequently, a reasonable SEM magnification should be recommended for the qualitative research of red clay microstructure. The optimum magnification range of SEM was from 1,500× to 4,000× or near 1,500× to 4,000×. In this article, SEM images with 2000× magnification were used for quantitative analysis.

#### Optimisation of threshold value

When SEM images are used to conduct microanalysis, the images require noise reduction, segmentation and binarisation, which converts the grey SEM image into the black-and-white image as follows^36^:

Assuming the greyscale of an SEM image was expressed as $f\left( {x,y} \right)$, $G$, where $G$ is the grey set of picture pixels $G$ = {0, 1, 2, …, 255}, then the image function can be defined as mapping: $f{:}M \times N \to G$, in which $M$ and $N$ are mapping parameters. Assuming a threshold $t$ ∈ $G$, $S = \left[ {S_{0} ,S_{1} } \right]$, where $S$ represents a binary grey level. The process of binarisation of an image is to transform it into an image function $f_{t} {:}M \times N \to S$, so that when $f\left( {x,y} \right) < t$, $f_{{\text{t}}} \left( {x,y} \right) = S_{0}$, in which $t$ is the threshold, instead, when $f\left( {x,y} \right) > t$, $f_{{\text{t}}} \left( {x,y} \right) = S_{1}$. Therefore, if the different threshold $t$ was used, the obtained results of image binarisation would be different, so it is necessary to find an optimal threshold $t$ for accurate results.

**Figure 19:**
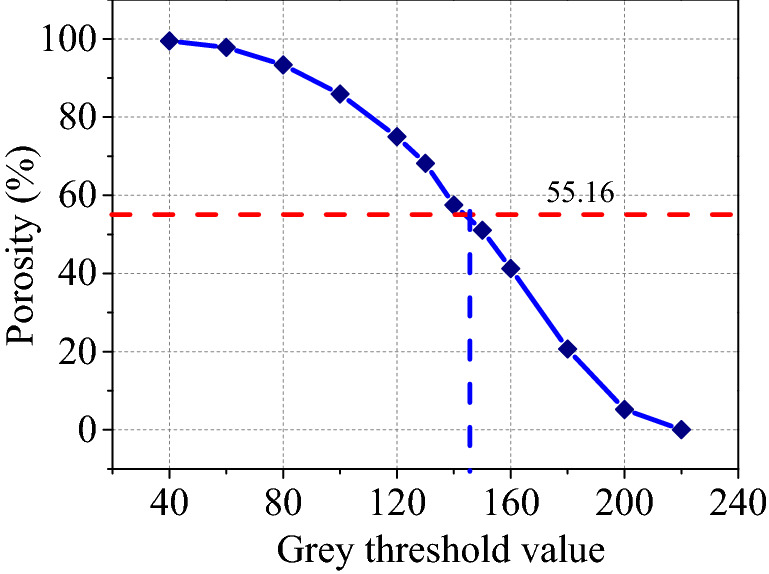
Influence of $t$ on porosity.

Figure 19 presents the influence of the grey threshold $t$ on the porosity from SEM images, showing that the obtained porosity from SEM images with 2000 × decreased with increasing grey threshold $t$ because for the same greyscale image when the threshold value became larger, the more distinct the target points become the black background colour, while the pixels originally representing soil particles may be mistaken for pores, thus increasing the number of pores. When the grey threshold $t$ was near 130, the obtained porosity was close to the test porosity, therefore, the optimal threshold value $t$ = 130 was used in this article.

#### Microstructure parameters of undisturbed and remoulded red clay

**Figure 20:**
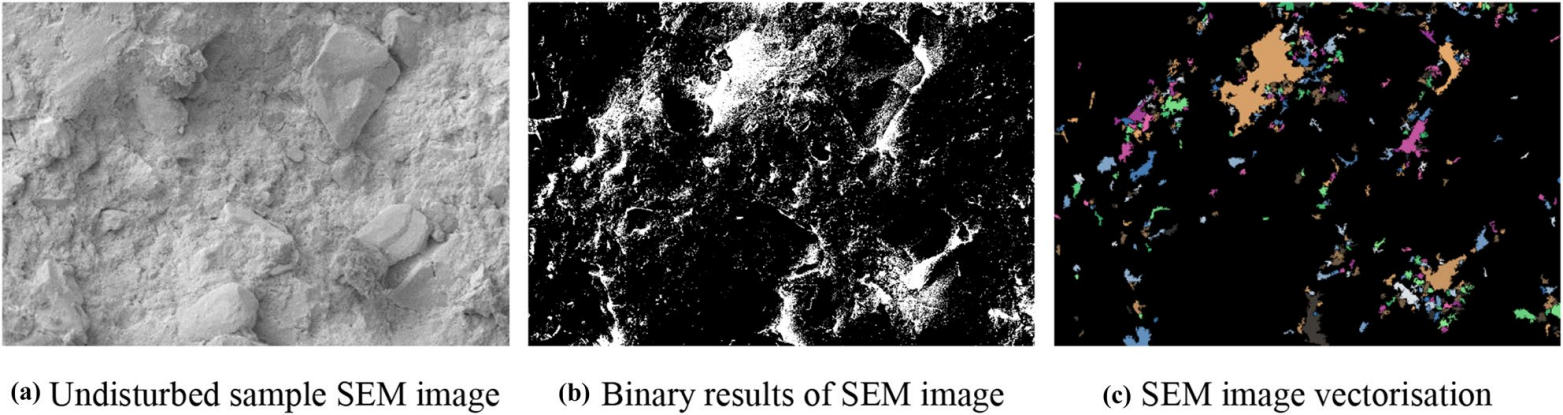
SEM images processing of Guiyang undisturbed red clay.

**Figure 21:**
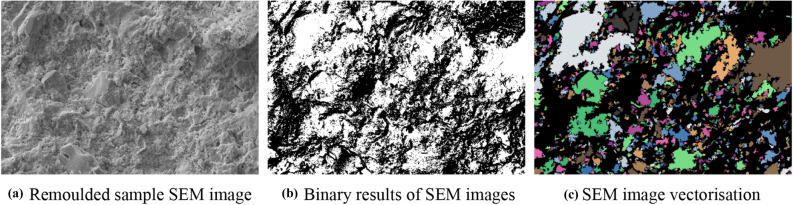
SEM image processing of Guiyang remoulded red clay.

The mechanical properties of soil are related to the shape, arrangement and distribution of pores, therefore, to investigate the multi-scale analysis of the microstructure characteristics of undisturbed and remoulded red clay from Guiyang based on the PCAS system, the pore parameters, average region area, average perimeter, average shape factor, maximum length, average width, probability entropy, area probability distribution index, the fractal dimension of pore distribution, and probability entropy, of the undisturbed and remoulded red clay were extracted. The processed SEM images after microanalysis are shown in Figs. 20 and 21. The extracted pore parameters of undisturbed and remoulded red clay are shown in Table 4. The parameters of probability entropy, probability distribution index and fractal dimension describe the direction, area distribution and shape factor change of pore system^39^. In terms of the soil pore shape, which is represented by the average shape factor, also called roundness, the parameter ranged from 0 to 1^36^. As the shape factor increased, the particle or pore gets closer to a circle. Conversely, with the decrease in the average shape factor, the pore shape becomes longer and narrower, thus the arrangement and combination of pores become more complex. The direction of the pore arrangement is expressed by probability entropy H^39^, which usually ranges from 0 to 1. When H is equal to 0, all pores are arranged in one direction, whereas when H is equal to 1, the direction of pores distribution is random. Therefore, when the probability entropy increases, the orientation directivity of pores become more complex.

**Table 4:** Pore parameters of undisturbed and remoulded red clay.

Parameters	Average region area	Average perimeter	Average shape factor	Maximum length	Average width	Probability entropy	Area probability distribution index	Fractal dimension of pore distribution
	(pixel)	(pixel)	–	(pixel)	(pixel)	–	–	–
Undisturbed	186.870	81.570	0.3895	250.090	12.790	0.9680	2.2763	3.024
Remoulded	358.290	92.320	0.3646	367.080	14.930	0.9729	2.1201	2.008

From Table 4, the fractal dimension of the pore distribution of remoulded red clay was less than that of undisturbed red clay. As the microstructure of remoulded soil was damaged after disturbance, the pores became uniform, with large pores becoming small and medium pores, so the fractal dimension of the pore distribution of remoulded soil is smaller than that of undisturbed soil^16^. The probability entropy of undisturbed red clay was also smaller than that of remoulded red clay. The lower the probability entropy, the higher the ordered degree of pore distribution^40^. The larger the probability index of distribution, the smaller the porosity^39^. The shape factor of undisturbed red clay was larger than that of remoulded red clay, indicating a more complex shape. Also, the pore area, perimeter, maximum length and average width of the undisturbed red clay were smaller than remoulded red clay, indicating that the red clay structure was damaged after disturbance, resulting in the increased pore area, as shown in Fig. 15.

## Conclusions

This study investigated the microstructure and mechanical properties of undisturbed and remoulded red clay from Guiyang, demonstrating the following:

1. Guiyang red clay has granular-shape, curved-slice and thin layer and flower-shaped ellipsoid aggregates, with point contact, line contact, surface contact and mosaic contact modes. There are X and Y-type cracks and pores in undisturbed red clay, which has flocculation, honeycomb and pseudosphere structures.
2. The quantitative analysis of the microstructure of red clay is influenced by the magnification and grey threshold and the recommended magnification ranged from 1,500 × to 4,000 × and the threshold values are near 130.
3. The deviatoric stress–strain curve of the undisturbed red clay from Guiyang exhibited obvious strain-softening behaviour, whereas the remoulded red clay exhibited strain hardening behaviour. For undisturbed red clay, the angle between the shear failure plane and the horizontal plane of the specimen was 60° when the specimen was destroyed, whereas the remoulded red clay exhibited a bulging failure. Also, the strength of undisturbed red clay was higher than that of remoulded red clay.
4. In comparison to remoulded red clay, the undisturbed red clay had a denser structure and exhibited obvious structural properties, with the pore distribution in one direction. Also, the pores of the undisturbed red clay were slit-shaped, whereas they were ordered circular-shaped in the remoulded red clay.

## Data Availability

All data, including image files, are available from the corresponding author on reasonable request.
