# Author Correction: Microscopic and mechanical properties of undisturbed and remoulded red clay from Guiyang, China

**DOI:** 10.1038/s41598-021-81993-z

**Published:** 2021-02-03

**Authors:** Yanzhao Zhang, Shaoyun Pu, Rita Yi Man Li, Jing Zhang

**Affiliations:** 1 College of Resource and Environmental Engineering, Guizhou University, Huaxi New Campus, Guiyang City, 550025 China; 2 Liyang City Construction Development Group Co., Ltd, Zhuzhou, 213300 China; 3 School of Transportation, Southeast University, Nanjing, 211189 Jiangsu China; 4 Sustainable Real Estate Research Centre, Hong Kong Shue Yan University, Hong Kong, China; 5 College of Geological Engineering and Geomatics, Chang’an University, Xi’an, 710054 Shaanxi China

Correction to: *Scientific Reports* https://doi.org/10.1038/s41598-020-71605-7, published online 22 October 2020

The original version of this Article contained an error in the title of the paper, where the word “undisturbed” was incorrectly given as “undistributed”. This has now been corrected in the PDF and HTML versions of the Article.

Additionally, the original version of this Article contained an error in the legend of Figure 5.

“Remoulded clay stress–strain curves^34^”.

now reads:

“Remoulded clay stress–strain curves^33^”.

Finally, the original version of this Article contained an error in the legend of Figure 6.

“Remoulded clay failure modes^34^”.

now reads:

“Remoulded clay failure modes^33^”.

